# Molecular characterization of Methicillin*-*resistant *Staphylococcus aureus* isolated in ready-to-eat food sold in supermarkets in Bobo-Dioulasso: case of charcuterie products

**DOI:** 10.1186/s12879-024-09603-7

**Published:** 2024-07-23

**Authors:** Namwin Siourimè Somda, Alima Mah Esther Traoré, Domonbabele François de Sales Hien, Yemah Bockarie, Abel Tankoano, Donatien Kaboré, Ouindgueta Juste Isidore Bonkoungou, Hagrétou Sawadogo-Lingani, Aly Savadogo

**Affiliations:** 1 Département Technologie Alimentaire, Institut de Recherche en Sciences Appliquées et Technologies (IRSAT), Direction Régionale de l’Ouest (DRO/Bobo-Dioulasso), 03 BP 2393, Bobo-Dioulasso, 03 Burkina Faso; 2 Département Technologie Alimentaire (DTA), Institut de Recherche en Sciences Appliquées et Technologies (IRSAT) 03 BP 7047, Ouagadougou, 03 Burkina Faso; 3 Département Entomologie Médicale et Parasitologie, Institut de Recherche en Sciences de la Santé (IRSS), Direction Régionale de l’Ouest (DRO/Bobo-Dioulasso), 01 BP 545, Bobo Dioulasso, 01 Burkina Faso; 4 Cape Coast Teaching Hospital, Ghana, Cape Coast, Interberton Road, P.O. Box: CT 1363, Cape Coast, Ghana; 5 Unité de Genomique et des Pathogènes One Health (UGenoPath-OH), Université Joseph Ki- Zerbo, UFR/SVT 03 BP 7021, Ouagadougou, 03 Burkina Faso; 6 Laboratoire de Biochimie et Immunologie Appliquée, Université Joseph Ki-Zerbo, UFR/SVT 03 BP 7021, Ouagadougou, 03 Burkina Faso

**Keywords:** MRSA, Charcuterie products, Supermarket, Enterotoxin genes, Bobo-Dioulasso

## Abstract

**Background:**

*Staphylococcus aureus (S. aureus)* is one of the most widespread bacterial pathogens in animals and humans, and its role as an important causative agent of food poisoning is well-documented. The aim of this study was to highlight and characterize the resistance patterns of methicillin-resistant *S. aureus* (MRSA) in charcuterie products sold in selected supermarkets (SM) in Bobo-Dioulasso, Burkina Faso.

**Methods:**

In this study, 72 samples including ham (n = 19), merguez (n = 22), sausage (n = 15) and minced meat (n = 16) were collected from 3 supermarkets. Standard microbiology methods were utilised to characterise *S. aureus* isolates. Phenotypic resistance patterns were investigated using the disk diffusion method on Mueller-Hinton agar. Genotypic testing using polymerase chain reaction (PCR) was performed on the isolates to detect the 16S-23S gene. Using specific primers, the following genes *PVL*,* TSST-1*,* mecA*,* gyrA*,* gyrB*,* qnrA*,* intI1 and aac(6’)-Ib-cr* were identified from purified DNA by PCR.

**Results:**

Among the 72 ready-to-eat food samples, *S. aureus* was present in 51, (70.83%). The yield was highest in both the ham and merguez food products, 15/51 (29.41%) each, followed by minced meat 12/51 (23.53%) and sausage 9/51 (17.65%). A total of 35 isolates (68.63%) were confirmed as *S. aureus* after molecular characterization using 16–23 S primers with 05 (14.29%) strains identified as MRSA. All of the MRSA and majority of the methicillin-sensitive *S.aureus* (MSSA) isolates were resistant to penicillin G, ampicillin, tetracycline and erythromycin, whereas one isolate from minced meat was found in SM3-harbouring *PVL*,* TSST-1*,* mecA*,* gyrA*,* gyrB* and *Int1* genes.

**Conclusions:**

Our study revealed a high prevalence of *S. aureus* in chacuterie products in Bobo-Dioulasso with antimicrobial profiles that show resistance to most antibiotics. These findings should inform and augment efforts to raise awareness among local supermarket owners on adequate food manufacturing practices as well as promoting food safety and hygiene.

## Background

*Staphylococcus aureus*, is an significant cause of foodborne diseases. Following a short incubation period, clinical episodes may be characterized by nausea, vomiting, lethargy, abdominal cramps or toxic shock syndrome [1]. Major food poisonings still occur in the modern world, especially in developing countries [2]. *S. aureus* is also implicated in diseases in humans and animals such as respiratory tract infections, nosocomial bacteremia, surgical site infections, cardiovascular infections, mastitis, dermatitis, and other suppurative conditions [3, 4]. The organism harbors inherent mechanisms that promote colonization, tissue damage, and infection and evasion of host defense mechanisms. Staphylococcal food poisoning is caused by contamination with the *S. aureus* enterotoxin [4, 5]. The wide-ranging toxic effects of this bacterium make staphylococcal foodborne infections a major public health concern. Moreover, its ability to show resistance to a wide range of antibiotics has led to limited therapeutic options for treating disease [6]. *S. aureus* shows resistance to most β-lactam antibiotics, linezolid, daptomycin, and vancomycin, which are the last-resort antibiotics for Gram-positive bacteria. The imprudent and indiscriminate usage of antibiotics in public and veterinary clinical practices has led to the development of multi-drug resistant pathogens [7]. For instance, β-lactam antibiotics are commonly used in the treatment of staphylococcal infections, but the number of *S. aureus* strains that secrete β-lactamase is increasing [8]. Contamination of food products by multi-drug resistant *S. aureus* strains has been linked to various factors such as poor preservation of food (breakage of the cold chain), culinary techniques, and handling of products during marketing [9, 10]. The investigation of *S. aureus* carriage among food handlers and analysis of the prevalence of toxin genes in colonizing strains is important to prevent food contamination with toxigenic strains that may be related to food poisoning or other diseases [10]. Nowadays, methicillin resistant *S. aureus* (MRSA) strains have been reported from various food sources, such as beef, milk, poultry, pork, vegetables as well as from environmental sources, suggesting that these are potential reservoirs [11, 12]. As is commonly known, foods have many different origins, and different types of MRSA are present in foods of different origin in different countries [13]. The presence of MRSA on food could be due to different manufacturing methods used, especially in developing countries, where food processing methods are generally traditional (food in direct contact with staff). The poor sanitary practices employed during cooking and sale of food multiply the risk of microbial contamination [14]. High ambient temperatures, especially in tropical environments have been described as a major factor responsible for facilitating the access and multiplication of bacterial contaminants in meat products [15]. In Burkina Faso, in recent years, many studies have shown a high prevalence of *S. aureus* isolated in clinical samples and resistance to methicillin [16–20]. However, to our knowledge, there is no local data on the prevalence and molecular characterization of MRSA from food and environmental sources. With the fight against antimicrobial resistance (AMR) now hinged on a robust adherence to the “One Health” strategy, it is highly relevant that data on MRSA is available from all areas that are potential reservoirs of *S. aureus*. In this regard, this study aims to (i) isolate *S. aureus* from selected charcuterie products, (ii) undertake molecular characterization of the isolates (*mecA* genes and other virulence genes) (iii) and describe the resistance profiles of the isolates to common antibiotics used.

## Methods

The study was carried out in Bobo-Dioulasso from April to December 2021. Three (3) supermarkets were used for sample collection. The charcuterie products utilized in this study were ham, merguez, sausages, and minced meat.

### *Samples collection*

Samples were collected according to the same conditions of purchase by the consumer. A total of 72 charcuterie samples were collected which included minced meat, ham, sausages and merguez sausages. These were composed of 18 samples of each product. Samples were packaged into labeled freezer bags in cooler containing cold accumulators under sterile conditions, and immediately transported to the Food Technology Department of Institute for Research in Applied Sciences and Technologies (DTA/IRSAT) for analysis.

### *Microbiological analysis*

Twenty-five (25) grams of each sample were added to 225 ml of Buffered Peptone Water (M028-500G, HIMEDIA, India) and homogenized using a stomacher (400 Circulator; Seward, London, UK). Ten-fold dilutions were obtained from the stock solution according to ISO:6887-2 methods [21]. The different samples prepared were inoculated on Baird Parker yellow agar supplemented with potassium tellurite. The inoculated Petri dishes were placed in an incubator previously cleaned with 65% alcohol and set at 37 °C ± 1.0 °C for 24 h ± 2 h for the incubation of *S. aureus*. All operations were carried out aseptically on a bench previously well cleaned with 65% alcohol. After the stipulated incubation time, characteristic colonies were introduced in Heart Infusion Broth (BHI) broths and then incubated at 37 °C for 24 h. The coagulase test was performed using freeze-dried rabbit plasma to differentiate *Staphylococcus aureus* strains from other *Staphylococcus* strains. To do this, five colonies were sub cultured in tubes containing Oxoid Brain BHI (7 ml) then incubated at 37 °C for 24 h. After incubation, 0.5 ml of the culture was added to 0.5 ml of rabbit plasma. The whole content was shaken well, then incubated at 37 °C. The tubes were examined after 4 h and then after 24 h. The formation of a clot was considered as coagulase reaction positive.

### Antibiotics resistance test

The phenotypic resistance profiles of *S. aureus* were investigated using the disk diffusion method on Mueller–Hinton agar (Merck, Germany) using swab according to procedure by the European Committee on Antimicrobial Susceptibility Testing [22]. Various antibiotics discs including penicillin G (10 µg/disk), ampicillin (10 µg/disk), amikacin (30 µg/disk), gentamicin (10 µg/disk)), ciprofloxacin (5 µg/disk), clindamycin (2 µg/disk), erythromycin (15 µg/disk), azithromycin (15 µg/disk), tetracycline (30 µg/disk) chloramphenicol (30 µg/disk), trimethoprim–sulfamethoxazole (25 µg/disk), cefoxitin (30 µg/disk), kanamycin (30 µg/disk) and rifampin (5 µg/disk) were used (Oxoid, UK). Three (3) Petri dishes were used for each sample (one dish for the cefoxitin, and for the remaining two Petri dishes, 7 antibiotic discs were used for each Petri dish). Multiresistance was defined as resistance to at least three different antibiotic families [23]. The process was completed using the protocol characterized previously. *S. aureus* (ATCC 43,300 and ATCC 29,213) was used as the quality control organism in antimicrobial susceptibility determination.

### Molecular detection

*Staphylococcus aureus* isolates were subjected to 16S-23S genomic analysis. The technique adopted was as described by Pantůček et al, [24] using primers G1 (5′-GAAGTCGTAACAAGG-3’) and L1 (5′-CAAGGCATCCACCGT-3’). The extraction of bacterial DNA was carried out by thermolysis releasing the bacterial DNA. Two to three well-isolated colonies from the bacterial culture were re-suspended in 300 µl of sterile distilled water. The mixture was heated in boiling water for 15 min. Then it was centrifuged at 12,000 rpm for 15 min. The supernatant was collected as a DNA template and stored at -20 °C for subsequent analyses. Afterwards, DNA was checked by nanodrop spectrophotometer for yield and purification and used for further analysis.

The screening of the different genes *PVL*,* TSST-1*,* mecA*,* gyrA*,* gyrB*,* qnrA*,* Int1* and *aac(6’)-Ib-cr* was carried out by PCR on the purified DNA using specific primers (Table 1). The reaction mixture was constituted for the different genes of 4 µl master mix; 0.5 µl forward primer (F); 0.5 µl reverse primer (R); 12.5 µl H_2_O PCR and 2.5 µl DNA for a total of 20 µl per PCR tube.

Once the PCR was complete for the migration of the amplicons, 10 µl of each amplicon were deposited in the wells on the agarose gel (1.5%) contained in a 0.5X TBE migration buffer) starting from the bottom to the top. A 100 bp molecular weight marker (Hyper Ladder1, Bioline) was used to evaluate the size of the different genes. The migration was carried out at 120 V, 300 mA using a voltage generator for 50 min to allow complete separation of the bands. After the migration of the amplicons, the gels were visualized in a Doc-print France device under ultraviolet (UV) light coupled with a printer (Digital Graphic Printer UP) and a computer equipped with a camera allowing photos of the bands.

**Table 1:** Primer sequences for virulence and antibiotic resistance genes

Genes	Primers (5’ – 3’)	Bp
*PVL*	F : AAATGCCACTGTTATCCAGAGGTA R : T TTGCAGCGTTTTGTTTTCG	433
*TSST-1*	F : ACCCCTGCCTTTCCATCATC R : T TTTCAGTATTTGTAACGCC	209
*mecA*	F : TCCAGGAATGCAGAAAGACC R : TCACCTGTTTGAGGGTGGAT	675
*gyrA*	F : TAC ACC GGT CAA CAT TGA GG R: TTA ATG ATT GCC GCC GTC GG	647
*gyrB*	F : TGA AAT GAC CCG CCG TAA AGG R : GCT GTG ATA ACG CAG TTT GTC CGGG	309
*Int1*	F : ACA TGT GAT GGC GAC GCA CGA R : AAT TCT GTC CTG GCT GGC GA	580
*qnrA*	F : TCA GCA AGA GGA TTT CTA R : GGC AGC ACT ATT ACT CCC	657
*aac(6’)-Ib-cr*	F : ATGACTGAGCATGACCTTGC R : TTAGGCATCACTGCGTGTTC	519

**Legend**: ***PVL***: Panton and Valentine leukotoxin, ***TSST-1***: Toxic shock syndrome toxin-1

### MRSA confirmation

Cefoxitin disks (30 µg) were used for detecting methicillin resistant isolates. Methicillin resistance was defined by a cefoxitin inhibition diameter < 27 mm for both MRSA. *S. aureus* ATCC 25,923 was used as a control. The *mecA/mecC* gene, which has been shown to confer methicillin resistance to *S. aureus* (MRSA), was also detected by PCR using primers as described previously.

### Data analysis

All data were presented as the mean ± standard deviation (SD). Statistical analyses were performed using R version 4.2.2 software. The effects of the origin, virulence gene, the resistance gene, and their interaction on the frequency of *Staphylococcus aureus* were analyzed, using a binomial Generalized Linear Model (GLM, glm function). For the model selection, the stepwise removal of terms was used, followed by likelihood ratio tests. Term removals that significantly reduced explanatory power were retained in the minimal adequate model. All the contamination rates were expressed with a 95% confident interval. Significance levels indicating statistical significance were denoted as follows: *p* < 0.05; **, *p* < 0.01; ***, *p* < 0.001; ****, *p* < 0.0001*****.

## Results

### Prevalence of coagulase positive *Staphylococcus* in ready-to-eat food

**Fig. 1:**
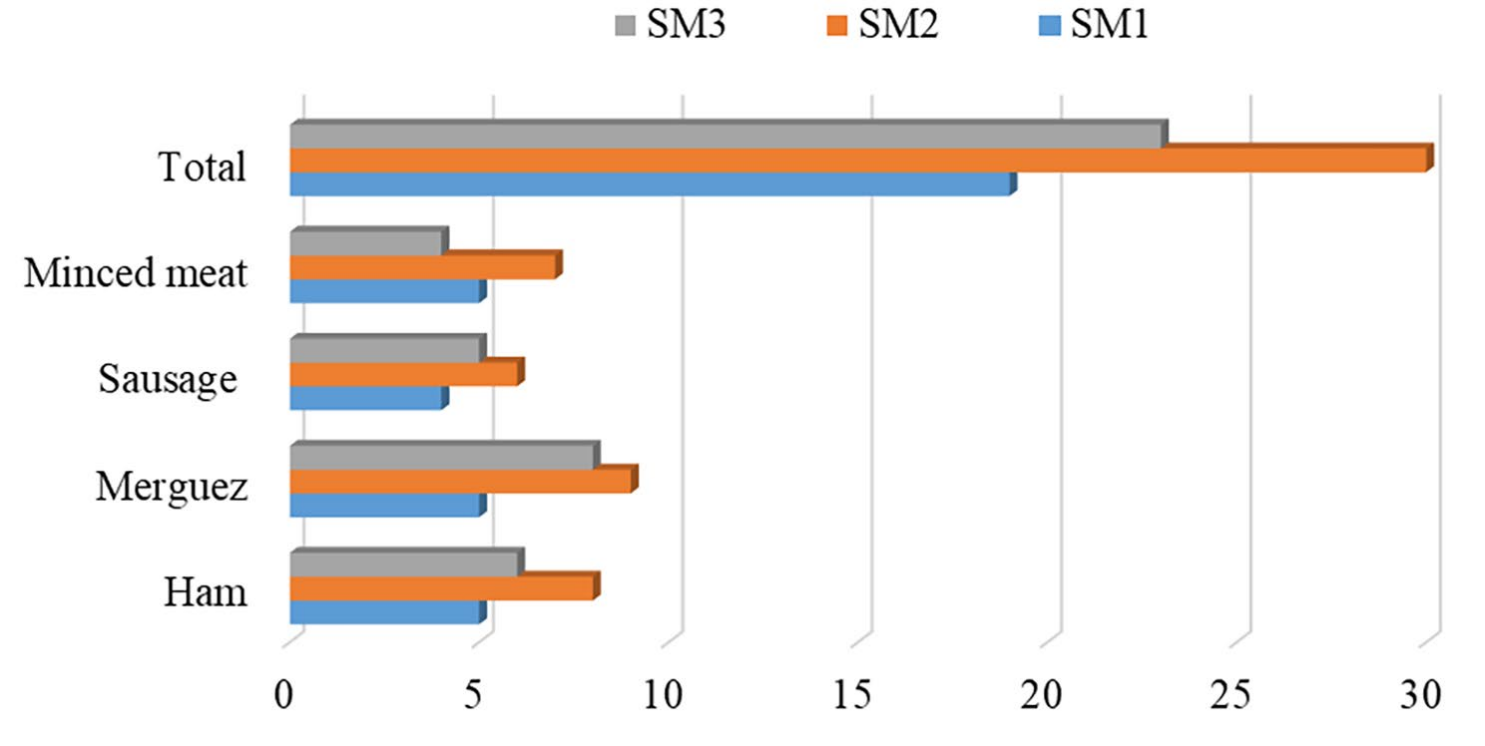
Distribution of samples according to supermarkets. Legend: SM1 = Super Market 1, SM2 = Super Market 2 and SM3 = Super Market 3

A total of 72 samples were collected from three (3) supermarkets (SM). These samples were composed 22 (30.56%) merguez, 19 (26.39%) ham, 16 (22.22%) minced meat and 15 (20.83%) sausage. According to SM, most samples were collected at SM2 (41.67%) followed by SM3 (31.94%) and SM1 (26.39%) (Fig. 1).

Among the 72 ready-to-eat food samples, 51 (70.83%) were positive for **coagulase positive *Staphylococcus*** according to microbiology standard methods used. This included 15/19 (78.95%) of ham samples, 15/22 (68.18%) of merguez, 12/16 (75%) of minced meat and 9/15 (60%) of sausage samples. Overall, the highest yield was obtained from the ham and merguez 15/51, (29.41%) for each product followed by minced meat 12/51 (23.53%), while the lowest yield was obtained from the sausage 9/51 (17.65%). This included 12/19 (63.16%) of the SM1 samples, 23/30 (76.67%) of the SM2 samples and 16/23 (69.57%) of the SM3 samples.

### Molecular characterization to *Staphylococcus aureus* confirmation using PCR with 16–23 S primers

**Fig. 2:**
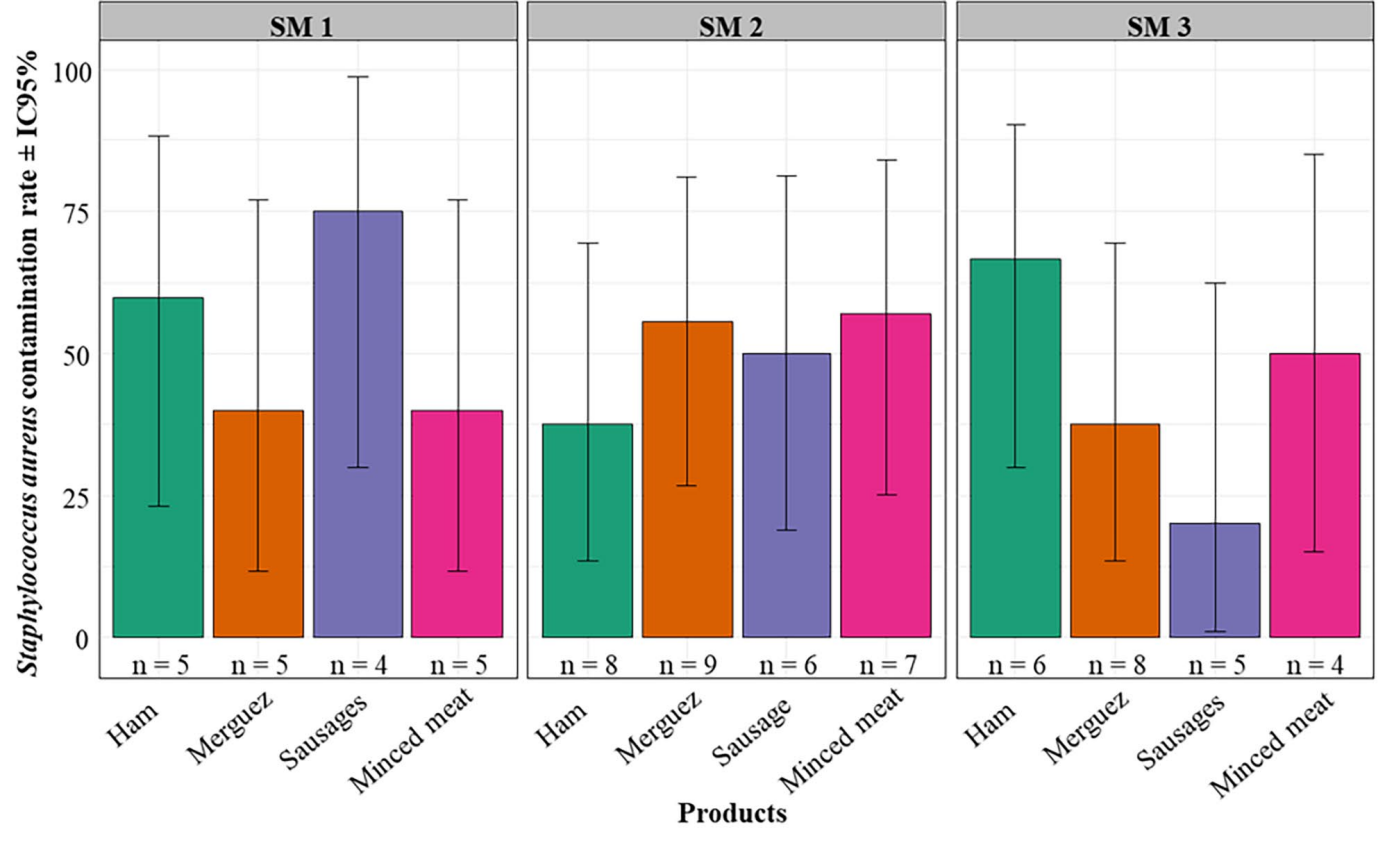
Prevalence of *Staphylococcus aureus* using PCR for confirmation. Legend: n = sample size (number) for each product per sites. There is no significant interaction between sites and products in the rate of product contamination. SM1 = Super Market 1, SM2 = Super Market 2 and SM3 = Super Market 3

In this study, 51 *Staphylococcus* spp. were isolated, among which, 35 isolates (68.63%) were confirmed as *S. aureus* after molecular characterization using 16–23 S primers. This included 10/19 (52.63%) of samples from SM1, 15/30 (50%) of samples from SM2 and 10/23 (43.48%) of samples from SM3. Overall, these confirmed *S. aureus* isolates were detected accordingly as follows: 10/19 (52.63%), 10/22 (45.45%), 7/15 (46.67%) and 8/16 (50%) from ham, merguez, sausage and minced meat respectively (Fig. 2). Binomial GLM analysis showed that, there was no significant interaction between sites and products in the rate of product contamination (*Chi2 = 4.8619; p = 0.5616).*

### Antimicrobial test and MRSA confirmation of *Staphylococcus aureus* isolates

**Fig. 3:**
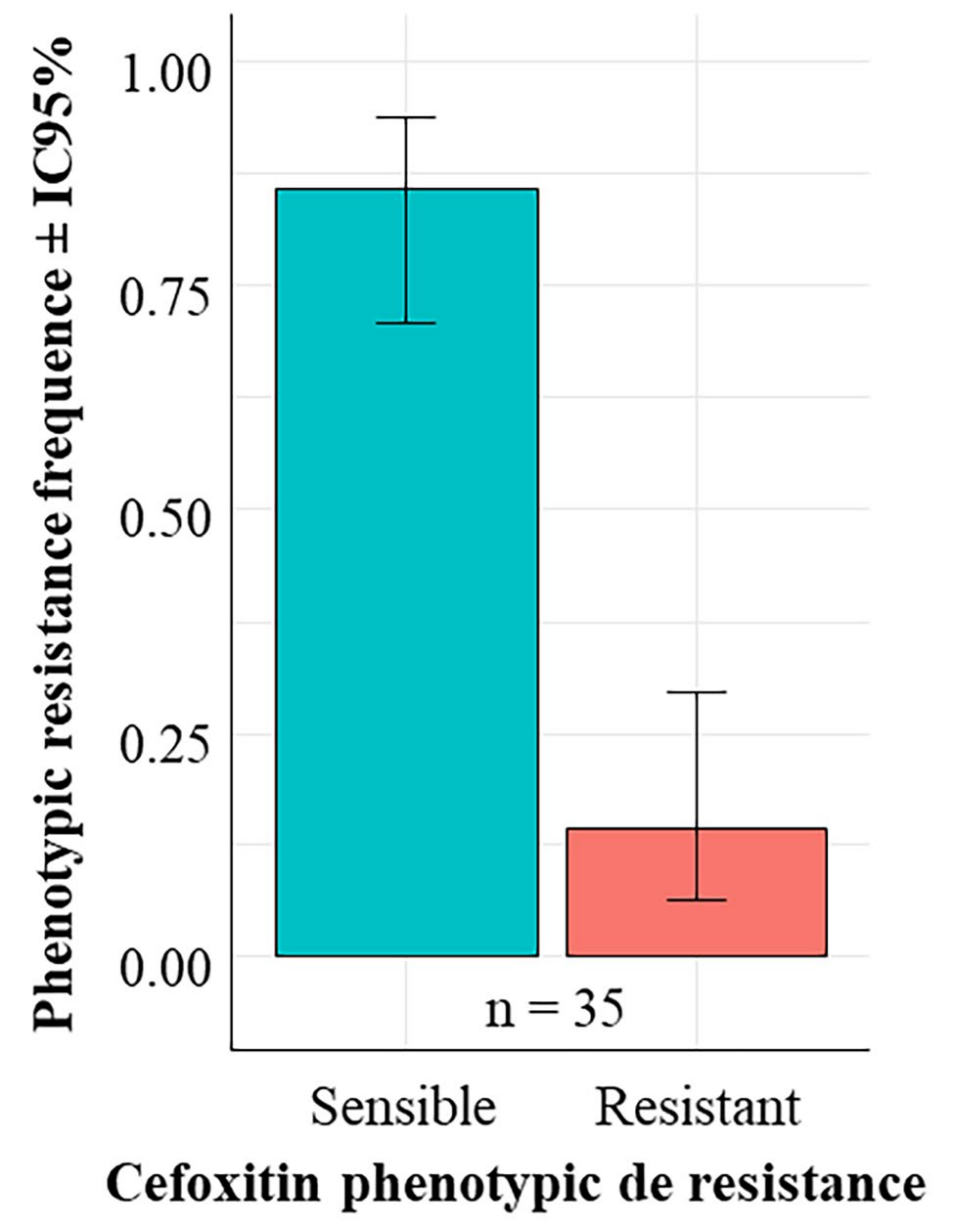
Prevalence of Methicillin Resistance of *Staphylococcus aureus* (MRSA). Legend: There is a significant difference in the frequency of phenotypes

Of the 35 *S. aureus* isolates recovered, 05 (14.29%) were confirmed as MRSA by cefoxitin disc diffusion test (Fig. 3). According to the binomial GLM analysis, there was no significant difference in the frequency of phenotypes (*Ch2 = 17.857; p = 2.381e-05*). These MRSA isolates were *mecA*-positive and resistant to penicillin G, ampicillin, tetracycline and erythromycin. Among them, 60% were resistant to clindamycin, azithromycin and chloramphenicol, and 40% were resistant to trimethoprim-sulfamethoxazole, rifampicin and kanamycin. All MRSA isolates were susceptible to gentamicin, ciprofloxacin and amikacin. The antimicrobial resistance profiles of the MRSA isolates are shown in Table 2.

All 30 methicillin-susceptible *S. aureus* (MSSA) isolates were resistant to at least one antimicrobial agent, while 25 isolates (83.33%) were resistant to at least three antimicrobials. The highest levels of resistance were observed for penicillin G (90%), tetracycline (86.67%), ampicillin (86.67%), azithromycin (80%) and erythromycin (73.33%). The antimicrobial resistance profiles of the MSSA strains are shown in Table 2.

**Table 2:** Antimicrobial resistance profiles of MRSA and MSSA strains isolated in charcuterie products

Antibiotics	Resistant of MRSA Numb (%)	Resistant of MSSA Numb (%)
Ampicillin (AMP, 10 µg)	5 (100)	26 (86.67)
Cefoxitin (FOX, 30 µg)	5 (100)	0
Penicillin G (P, 10 µg)	5 (100)	27 (90)
Chloramphenicol (C, 30 µg)	3 (60)	15 (50%)
Tetracycline (TE, 30 µg)	5 (100)	26 (86.67)
Ciprofloxacin (CIP, 5 µg)	0	0
Amikacin (AK, 30 µg)	0	0
Gentamicin (CN, 10 µg)	0	0
Azithromycin (AZT, 15 µg)	3 (60)	24 (80)
Trimethoprim-Sulfamethoxazole (SXT, 25 µg)	2 (40)	15 (50%)
Erythromycin (E, 15 µg)	5 (100)	22 (73.33)
Clindamycin (DA, 2 µg)	3 (60)	12 (40)
Rifampicin (RD, 5 µg)	2 (40)	10 (33.33)
Kanamycin (K, 30 µg)	2 (40)	5 (16.67)
Total Numb of strains	5 (100)	30 (100)

**Legend**: Number (Numb), Percent (%). We have presented the same table for the two types of Staphylococcus (MRSA and MSSA). The second column corresponds to MRSA (5 Strains). The third column corresponds to the MSSA (30 strains). Additionally, among these strains there are others which are resistant to certain antibiotics

### Molecular characterization of toxin production

**Fig. 4:**
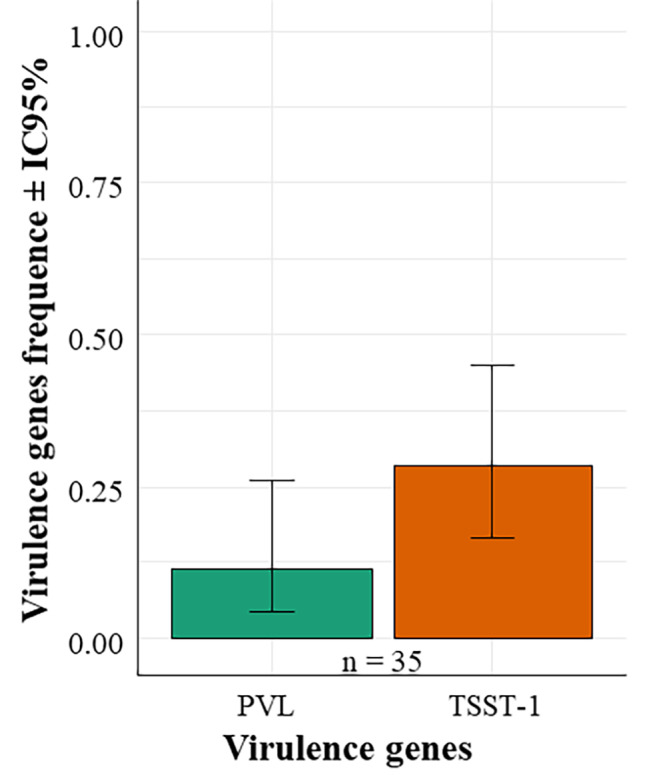
Distribution of virulence genes. Legend: n = size (number) of the sample tested. There is no significant difference in the frequency of virulence genes. PVL = Panton Valentine Leukocidin. TSST-1 = toxic-shock syndrome toxin-1

Out of the 35 isolates, genes coding for Panton and Valentine leukotoxin (*PVL*) were found in 4 isolates (11.43%) and Toxic shock syndrome toxin-1 *(TSST-1)* in 10 isolates (28.57%) (Table 3). Two strains harbored both *PVL* and *TSST-1*genes. Among strains positive for the *PVL* gene, two (2) harbored the *mecA* gene while 3 *TSST-1* positive strains had the *mecA* gene. However, two strains harbored multiple genes - *PVL*,* TSST-1* and *mecA* (Table 3). These strains were isolated in merguez (SM1) and minced meat (SM3) Fig. 4. According to binomial GLM analysis, there was no significant difference in the frequency of virulence genes (*Chi2 = 3.3007; p = 0.06925*).

### Molecular characterization of resistance gene

**Fig. 5:**
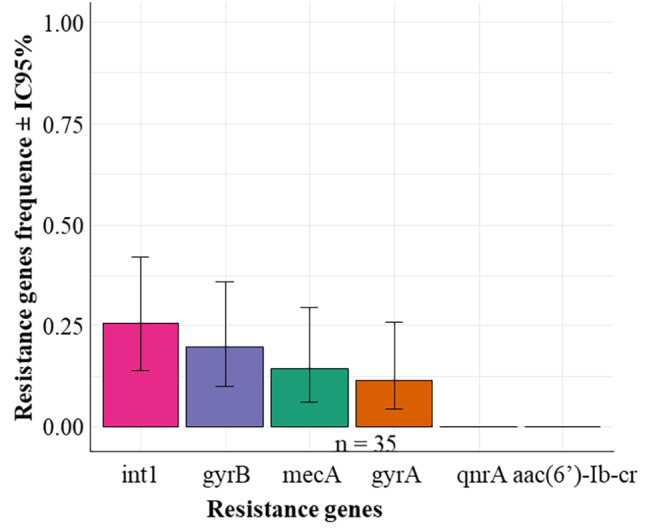
Distribution of Resistance genes. Legend: n = size (number) of the sample tested. There is no significant difference in the frequency of resistance genes

The use of PCR enabled detection of antibiotic resistance genes in the isolated strains. Among 35 isolates, 4 (11.43%) harbored *gyrA*, 7 (20%) *gyrB* and 9 (25.71%) *Int1* (Table 3). However, *qnrA* and *aac(6’)-Ib-cr* were not detected (Fig. 5). There was no significant difference in the frequency of resistance genes (*Chi2 = 24.793; p = 0.0001527*). One strain of *S. aureus* isolated from minced meat at SM3 carried the following genes *PVL*,* TSST-1*,* mecA*,* gyrA*,* gyrB* and *Int1*. Similarly, two strains harbored *PVL*,* TSST-1*,* mecA*,* gyrB* and *Int1*genes. *Int1* gene was detected from all MRSA; these strains were isolated in two merguez samples, 2 ham samples and one minced meat sample.

**Table 3:** Prevalence of virulence and resistance genes in *S. Aureus* isolated from charcuterie products in Bobo-Dioulasso

SM	Products	16–23 S	PVL	TSST-1	mecA	gyrA	gyrB	int1	cefoxitin *R*
**SM1**	Ham	3	0	1	0	0	1	0	0
	Merguez	2	1	1	1	0	2	1	1
	Sausage	3	0	1	0	0	0	0	0
	Minced meat	2	0	0	0	1	0	1	0
**SM2**	Ham	3	0	1	1	2	0	2	1
	Merguez	5	0	1	1	0	2	1	1
	Sausage	3	0	1	0	0	0	1	0
	Minced meat	4	1	0	0	0	0	1	0
**SM3**	Ham	4	0	2	1	0	0	1	1
	Merguez	3	1	1	0	0	1	0	0
	Sausage	1	0	0	0	0	0	0	0
	Minced meat	2	1	1	1	1	1	1	1
**Total**		35/51(68.63%)	4/51(7.84%)	10/51(19.61%)	5/51(9.80%)	4/51(7.84%)	7/51(13.73%)	9/51(17.65%)	5/51(9.80%)

**Legend**: ***PVL***: Panton and Valentine leukotoxin, ***TSST-1***: Toxic shock syndrome toxin-1, **SM**: Super Market,

## Discussion

Overall, the microbiological quality of the food products analyzed was sub-optimal given the results (51/72) of this study among which 35 MRSA isolates were detected. This finding could potentially be due to inadequate food-handling training and skills of frontline staff at the supermarkets visited, as well as poor conditions of storage and sale of charcuterie products. This finding may also have been exacerbated by sub-optimal enforcement of standard hygienic practices in the sales environment especially in circumstances when the sales staff are overwhelmed by the urgency to meet the demand of several people being attended to simultaneously. Many studies on food-based microbiological characterization have detected the presence of MRSA in foods. In some of these studies, [25–28], MRSA was found in 84.8% in broiler’s samples, 35% in raw beef, 67.5% in different food stings and 9.89% in raw retail meat samples respectively. It is known that *S. aureus* transmission is most often potentiated by poor staff hygiene practices as these organisms colonize the skin and mucous membranes, particularly on the hands and nasal cavity. *S. aureus* can also adhere to the gloves of employees in food processing establishments and therefore, can be transmitted to other humans or to the environment if gloves are not changed frequently. Other researchers have stated that contaminations can occur at the level of the sale materials, the seller and the likelihood of biofilms developing in deli meats [29]. Additionally, it has been shown that certain bacteria such as *Enterobacteriaceae* and certain species of *Staphylococcus* colonize the respiratory tract of infected people, as well as inanimate surfaces such as bedding, clothing, work and kitchen equipment and doorknobs, thereby increasing the risk of contamination of food processing environments [25]. When meat contaminated with *S. aureus* is undercooked or stored at inappropriate temperatures, toxins accumulate and with ingestion, this may lead to staphylococcal food poisoning [30]. This is further exacerbated in settings where the raw materials resulting from the processing of these products are not sold on site but transported from one city to another in adverse conditions such as improper maintenance of the cold chain during transport [30, 31]. All these factors contribute to food contamination, proliferation and propagation.

All the MRSA and many of the MSSA isolates were resistant to at least three drugs. The relatively high prevalence of these multi-drug resistant strains among ready-to-eat food products sold in supermarkets in Burkina Faso is alarming and of grave public health concern. Effective interventions need to be in place to tackle and track the potential transmission of multi-resistant strains as spread is likely to occur if standard food hygiene and safety measures are not enforced across food distribution networks within the country. Our study has shown that all the MRSA and majority of the MSSA isolates were resistant to penicillin G, ampicillin, tetracycline and erythromycin. These results are similar to findings of previous studies done on meat and other types of foods [13, 32]. One possible explanation of this finding may be related to the food source. This is because most animal or animal product derived food-related *S. aureus* originate from animal farms that used these antibiotics as food supplements in animal feed [11]. Some charcuterie products are made from pork. Furthermore, according to Wall et al., [33], the intensive conditions under which pigs are housed during production are a risk factor for spreading disease, resulting in high antibiotic use to control and treat infections. Similarly, the increasing routine use of antibiotics as growth promoters for prophylaxis, metaphylaxis, and treatment continues to have a profound devastating effect on antimicrobial resistance control as selective pressure elaborates and escalates resistance [23].

*S. aureus* harbors various virulence determinants that contribute to its pathogenicity. Therefore, animal foods may be a source of transmission of pathogenic strains in production facilities to humans and the environment [34]. Toxins constitute an important group of *S. aureus* virulence factors [35], with toxins being important to food safety. In this study, 28.57% food-related MRSA isolates harbored *TSST-1* genes and 11.43%, *PVL* genes (other toxin) among which, two isolates harbored both genes *PVL* and *TSST-1*. Our results are consistent with the results of other studies, which show a low presence of these genes in *S. aureus* isolates. This is the case for [36–38]. However, a substantial number of *S. aureus* isolates carried the *PVL* and *TSST-1* gene according to [32, 39] in pig and pig workers from Nigeria and South Africa. This difference could be explained by the fact that our studies focused on ready-to-eat foods. It has been demonstrated by some authors that pork are reservoirs of these virulent *S. aureus* [32, 39, 40].

In this study, it was found that certain strains of MRSA harbored virulence genes and certain antibiotic resistance genes. One isolate harbored the genes - *mecA*,* PVL*,* TSST-1*,* gyrA*,* gyrB*,* qnrA* and *Int1*, a mobile genetic element, which is responsible for the dissemination of resistance genes. Consumption of food contaminated by this isolate may potentially be a serious problem for the consumer and those around them because there is a risk of propagation. Integrons are elements of resistance gene transfer. MRSAs that harbor them can easily transfer resistance genes to other bacteria, which could make it difficult to treat certain *S. aureus* infections. However, the findings of our study are even more alarming in relation to public health considerations as ready-to-eat (RTE) foods are consumed without further cooking, which would have eliminated or reduced the microbial load. Consequently, the incidence of *S. aureus* and MRSA in RTE foods, along with the spread of antibiotic resistant strains, represents a potential health hazard to humans.

The findings in this study would raise awareness among supermarket owners in Bobo-Dioulasso, so that optimal food manufacturing, safety and hygiene practices can be enforced. Furthermore, these findings would inform public health practitioners including health and sanitation officials and community town hall workers to take necessary measures to guarantee food safety for the general public. Findings from this study will also provide local data to relevant authorities on *S. aureus* prevalence and resistance profiles in food products in the fight against antimicrobial resistance.

Limitation of study: A limiting factor of this study was the inability to sequence PCR products and this was largely due to inadequate finances. Secondly, the authors lacked resources to carry out multilocus sequence typing (MLST) and pulsed-field gel electrophoresis (PFGE) for determination of clones of isolates.

## Conclusion

Our study highlighted that *S. aureus* contamination is high in charcuterie products sold in supermarkets at Bobo-Dioulasso. Among these isolates, those with MRSA were highly resistant to common antibiotics. In our study, *S. aureus* strains contained genes producing toxins *PVL* and *TSST-1* which suggest pathogenicity as well as harboring genes that confer multi-resistance. This food contamination poses a high risk of food poisoning to consumers with a potentially difficult-to-treat illness course for those who succumb to disease. There is also a dire need for proper hygienic conditions to be instituted in production areas for charcuterie products as well as in the sales environment. Advocacy and capacity building on good food processing and safety practices for the supermarket workforce in Bobo-Dioulasso is mandatory.

## Data Availability

Data supporting the finding of this research are available upon the reasonable request from the corresponding author.

## Abbreviations

AMR: Antimicrobial Resistant
BHI: Oxoid Brain Heart Infusion Broth
MRSA: Methicillin-Resistant Staphylococcus aureus
MSSA: Methicillin-Sensitive Staphylococcus aureus
PVL: Panton and Valentine leukotoxin
TSST-1: Toxic shock syndrome toxin-1
ISRAT/DTA: Institut de Recherche en Sciences Appliquées et Technologies / Département Technologie Alimentaire
IRSS: Institut de Recherche en Sciences de la Santé
SM: Super Market
RTE: Ready-to-eat

## References

[1] Wang L, Ruan S. Modeling nosocomial infections of methicillin-resistant *Staphylococcus aureus* with environment contamination. Sci Rep. 2017;7(1):580. 10.1038/s41598-017-00261-1. 28373644 10.1038/s41598-017-00261-1 PMC5428062

[2] Assogba MHM, Salifou CFA, Tobada P, Aboudou AK, Bakary AB, Dahouda M, et al. Impact De La rupture de la chaîne de froid sur la qualité microbiologique de Scomber scombrus (maquereau commun) et de Trachurus trachurus (chinchard) dans le Sud Du Bénin. Int J Innov Appl Stud. 2018;24(2):623–32. http://www.ijias.issr-journals.org/.

[3] Asante J, Hetsa BA, Amoako DG, Abia ALK, Bester LA, Essack SY. Multidrug-resistant coagulase-negative staphylococci isolated from bloodstream in the uMgungundlovu District of KwaZulu-Natal Province in South Africa: emerging pathogens. Antibiotics. 2021;10(2):198. 10.3390/antibiotics10020198. 33670659 10.3390/antibiotics10020198 PMC7922184

[4] Oogai Y, Matsuo M, Hashimoto M, Kato F, Sugai M, Komatsuzawa H. Expression of virulence factors by *Staphylococcus aureus* grown in serum. Appl Environ Microbiol. 2011;77(22):8097–105. 10.1128/AEM.05316-11. 21926198 10.1128/AEM.05316-11 PMC3208999

[5] Papadopoulos P, Papadopoulos T, Angelidis AS, Kotzamanidis C, Zdragas A, Papa A, et al. Prevalence, antimicrobial susceptibility and characterization of Staphylococcus aureus and methicillin-resistant *Staphylococcus aureus* isolated from dairy industries in north-central and north-eastern Greece. Int J Food Microbiol. 2019;291:35–41. 10.1016/j.ijfoodmicro.2018.11.007. 30445283 10.1016/j.ijfoodmicro.2018.11.007

[6] Foster TJ. Antibiotic resistance in *Staphylococcus aureus* current status and future prospects. FEMS Microbiol Rev. 2017;41(3):430–49. 10.1093/femsre/fux007. 28419231 10.1093/femsre/fux007

[7] Aqib AI, Ijaz M, Anjum AA, Malik MAR, Mehmood K, Farooqi SH, et al. Antibiotic susceptibilities and prevalence of Methicillin resistant *Staphylococcus aureus* (MRSA) isolated from bovine milk in Pakistan. Acta Trop. 2017;176:168–72. 10.1016/j.actatropica.2017.08.008. 28797802 10.1016/j.actatropica.2017.08.008

[8] Guardabassi L, O’Donoghue M, Moodley A, Ho J, Boost M. Novel lineage of methicillin-resistant *Staphylococcus aureus*, Hong Kong. Emerg Infect Dis. 2009;15(12):1998. 10.3201/eid1512.090378. 19961685 10.3201/eid1512.090378 PMC3044525

[9] Zehra A, Gulzar M, Singh R, Kaur S, Gill J. Prevalence, multidrug resistance and molecular typing of methicillin-resistant *Staphylococcus aureus* (MRSA) in retail meat from Punjab, India. J Glob Antimicrob Resist. 2019;16:152–8. 10.1016/j.jgar.2018.10.005. 30312831 10.1016/j.jgar.2018.10.005

[10] Aung MS, San T, Aye MM, Mya S, Maw WW, Zan KN, et al. Prevalence and genetic characteristics of *Staphylococcus aureus* and *Staphylococcus Argenteus* isolates harboring Panton-Valentine leukocidin, enterotoxins, and TSST-1 genes from food handlers in Myanmar. Toxins. 2017;9(8):241. 10.3390/toxins9080241. 28777321 10.3390/toxins9080241 PMC5577575

[11] Wang X, Li G, Xia X, Yang B, Xi M, Meng J. Antimicrobial susceptibility and molecular typing of methicillin-resistant *Staphylococcus aureus* in retail foods in Shaanxi, China. Foodborne Pathog Dis. 2014;11(4):281–6. 10.1089/fpd.2013.1643. 24404781 10.1089/fpd.2013.1643

[12] Wu S, Huang J, Wu Q, Zhang F, Zhang J, Lei T, et al. Prevalence and characterization of *Staphylococcus aureus* isolated from retail vegetables in China. Front Microbiol. 2018;9:1263. 10.3389/fmicb.2018.01263. 29963025 10.3389/fmicb.2018.01263 PMC6011812

[13] Wu S, Huang J, Zhang F, Wu Q, Zhang J, Pang R, et al. Prevalence and characterization of food-related methicillin-resistant *Staphylococcus aureus* (MRSA) in China. Front Microbiol. 2019;10:304. 10.3389/fmicb.2019.00304. 30842766 10.3389/fmicb.2019.00304 PMC6391343

[14] Somda NS, Bonkoungou OJ, Zongo C, Kagambèga A, Bassolé IH, Traoré Y, et al. Safety of ready-to‐eat chicken in Burkina Faso: microbiological quality, antibiotic resistance, and virulence genes in *Escherichia coli* isolated from chicken samples of Ouagadougou. Food Sci Nutr. 2018;6(4):1077–84. 10.1002/fsn3.650. 29983972 10.1002/fsn3.650 PMC6021723

[15] Barro N, Bello AR, Savadogo A, Ouattara CAT, Iiboudo A. Hygienic status assessment of dish washing waters, utensils, hands and pieces of money from street food processing sites in Ouagadougou (Burkina Faso). Afr J Biotechnol 2006, 5(11). http://www.academicjournals.org/AJB.

[16] Traoré R, Ouédraogo GA, Ouédraogo AS, Savadogo A, Zongo C, Godreuil S. News sequences types of *Staphylococcus aureus* isolated from human pathologicals fluids in Burkina Faso. BMC Res Notes. 2024;17(1):151. 10.1186/s13104-024-06805-9. 38831376 10.1186/s13104-024-06805-9 PMC11145786

[17] Sore S, Sawadogo Y, Sanou S, Beogo S, Dakouo S, Djamalladine M. Portage nasal de *Staphylococcus aureus* résistant à La méticilline Chez Des Volontaires Sains et des malades hospitalisés à Ouagadougou. Burkina Faso. 2020. 10.53597/remim.v15i2.1734. 10.53597/remim.v15i2.1734

[18] Ouedraogo A-S, Dunyach-Remy C, Kissou A, Sanou S, Poda A, Kyelem CG, et al. High nasal carriage rate of *Staphylococcus aureus* containing panton-valentine leukocidin-and EDIN-encoding genes in community and hospital settings in Burkina Faso. Front Microbiol. 2016;7:1406. 10.3389/fmicb.2016.01406. 27679613 10.3389/fmicb.2016.01406 PMC5020597

[19] Ba AK, Diendere A, Sanou M, Diallo I, Tamini LT, Benin A, et al. Résistance aux antibiotiques des souches de *Staphylococcus aureus* et des enterobactéries isolés Au LNSP De Ouagadougou (Burkina Faso). Sci et Technique Sci de la Santé. 2019;42(1):83–94.

[20] Guira O, Tiéno H, Traoré S, Diallo I, Ouangré E, Sagna Y, et al. Écologie bactérienne et facteurs déterminant le profil bactériologique Du Pied diabétique infecté à Ouagadougou (Burkina Faso). Bull Soc Pathol Exot. 2015;108:307–11. 10.1007/s13149-015-0442-5. 26187771 10.1007/s13149-015-0442-5

[21] ISO6887-2. Préparation des échantillons, de la suspension mère et des dilutions écimales en vue de l’examen microbiologique. 2004, V08-010-2 16.

[22] EUCAST: Breakpoint tables for interpretation of MICs and zone diameters. 2020:18 p.

[23] Magiorakos A-P, Srinivasan A, Carey RB, Carmeli Y, Falagas M, Giske C, et al. Multidrug-resistant, extensively drug-resistant and pandrug-resistant bacteria: an international expert proposal for interim standard definitions for acquired resistance. Clin Microbiol Infect. 2012;18(3):268–81. 10.1111/j.1469-0691.2011.03570.x. 21793988 10.1111/j.1469-0691.2011.03570.x

[24] Pantůček R, Sedláček I, Petráš P, Koukalová D, Švec P, Štětina V et al. *Staphylococcus simiae* sp. nov., isolated from south American squirrel monkeys. Int J Syst Evol Microbiol *2*005, 55(5):1953–8. 10.1099/ijs.0.63590-0. 10.1099/ijs.0.63590-0 16166694

[25] Shahid AH, Nazir KNH, El Zowalaty ME, Kabir A, Sarker SA, Siddique MP, et al. Molecular detection of Vancomycin and methicillin resistance in *Staphylococcus aureus* isolated from food processing environments. One Health. 2021;13:100276. 10.1016/j.onehlt.2021.100276. 34409147 10.1016/j.onehlt.2021.100276 PMC8361190

[26] Assafi MS, Hado HA, Abdulrahman IS. Detection of methicillin-resistant *Staphylococcus aureus* in broiler and broilers farm workers in Duhok, Iraq by using conventional and PCR techniques. Iraqi J Vet Sci. 2020;34(1):15–22. 10.33899/ijvs.2019.125757.1145

[27] Torki Baghbaderani Z, Shakerian A, Rahimi E. Phenotypic and genotypic assessment of antibiotic resistance of Staphylococcus aureus bacteria isolated from retail meat. Infect Drug Resist. 2020;1339–49. 10.2147/IDR.S241189. 10.2147/IDR.S241189 PMC7213866 32440171

[28] Dorjgochoo A, Batbayar A, Tsend-Ayush A, Erdenebayar O, Byambadorj B, Jav S, et al. Detection of virulence genes of *Staphylococcus aureus* isolated from raw beef for retail sale in the markets of Ulaanbaatar city, Mongolia. BMC Microbiol. 2023;23(1):372. 10.1186/s12866-023-03122-2. 38031000 10.1186/s12866-023-03122-2 PMC10685515

[29] Salamandane A, Correia J, Muetanene BA, dos Santos M, Malfeito-Ferreira M, Brito L. Methicillin Resistance of Food-Borne Biofilm-forming Staphylococci. Appl Sci. 2023;13(13):7725. 10.3390/app13137725. 10.3390/app13137725

[30] Kluytmans J. Methicillin-resistant *Staphylococcus aureus* in food products: cause for concern or case for complacency. Clin Microbiol Infect. 2010;16(1):11–5. 10.1111/j.1469-0691.2009.03110.x. 20002686 10.1111/j.1469-0691.2009.03110.x

[31] Somda NS, Kaboré D, Tankoano A, Somda MK, Ouattara A, Paré A, et al. Antimicrobial resistance of *Staphylococcus aureus* and *Pseudomonas* spp. isolated from coated skewers sold in Ouagadougou, Burkina Faso. J Food Saf Hyg. 2021;7(4):237–47. http://jfsh.tums.ac.ir.

[32] Sineke N, Asante J, Amoako DG, Abia ALK, Perrett K, Bester LA, et al. *Staphylococcus aureus* in intensive pig production in South Africa: antibiotic resistance, virulence determinants, and clonality. Pathogens. 2021;10(3):317. 10.3390/pathogens10030317. 33800367 10.3390/pathogens10030317 PMC8000748

[33] Wall B, Mateus A, Marshall L, Pfeiffer D, Lubroth J, Ormel H et al. Drivers, dynamics and epidemiology of antimicrobial resistance in animal production. FAO 2016. https://rvc-repository.worktribe.com/preview/1398981/10602.pdf.

[34] Courvalin P. Vancomycin resistance in gram-positive cocci. *Clin. Infect. Dis* 2006, 42(*Supplement_1*):S25-S34. 1058–4838/2006/4201S1-0005$15.00. 10.1086/491711 16323116

[35] Yang X, Wu Q. Multilocus sequence typing and virulence-associated gene profile analysis of *Staphylococcus aureus* isolates from retail ready-to-eat food in China. Front Microbiol. 2018;9:329112. 10.3389/fmicb.2018.00197. 10.3389/fmicb.2018.00197 PMC5890145 29662467

[36] Li H, Andersen PS, Stegger M, Sieber RN, Ingmer H, Staubrand N, et al. Antimicrobial resistance and virulence gene profiles of methicillin-resistant and-susceptible *Staphylococcus aureus* from food products in Denmark. Front Microbiol. 2019;10:493345. 10.3389/fmicb.2019.02681. 10.3389/fmicb.2019.02681 PMC6920630 31920996

[37] Hammad AM, Watanabe W, Fujii T, Shimamoto T. Occurrence and characteristics of methicillin-resistant and-susceptible *Staphylococcus aureus* and methicillin-resistant coagulase-negative staphylococci from Japanese retail ready-to-eat raw fish. Int J Food Microbiol. 2012;156(3):286–9. 10.1016/j.ijfoodmicro.2012.03.022. 22541390 10.1016/j.ijfoodmicro.2012.03.022

[38] Monecke S, Coombs G, Shore AC, Coleman DC, Akpaka P, Borg M, et al. A field guide to pandemic, epidemic and sporadic clones of methicillin-resistant *Staphylococcus aureus*. PLoS ONE. 2011;6(4):e17936. 10.1371/journal.pone.0017936. 21494333 10.1371/journal.pone.0017936 PMC3071808

[39] Neyaz L, Wells H, Fakhr MK. Molecular characterization of *Staphylococcus aureus* plasmids associated with strains isolated from various retail meats. Front Microbiol. 2020;11:492997. 10.3389/fmicb.2020.00223. 10.3389/fmicb.2020.00223 PMC7042431 32140145

[40] Kimera ZI, Frumence G, Mboera LE, Rweyemamu M, Mshana SE, Matee MI. Assessment of drivers of antimicrobial use and resistance in poultry and domestic pig farming in the Msimbazi river basin in Tanzania. Antibiotics. 2020;9(12):838. 10.3390/antibiotics9120838. 33255152 10.3390/antibiotics9120838 PMC7760815

